# Physicochemistry and cardiovascular toxicity of metal fume PM_2.5_: a study of human coronary artery endothelial cells and welding workers

**DOI:** 10.1038/srep33515

**Published:** 2016-09-19

**Authors:** Chane-Yu Lai, Ching-Huang Lai, Hsiao-Chi Chuang, Chih-Hong Pan, Cheng-Chieh Yen, Wen-Yi Lin, Jen-Kun Chen, Lian-Yu Lin, Kai-Jen Chuang

**Affiliations:** 1 Department of Occupational Safety and Health, Chung Shan Medical University, Taichung, Taiwan; 2 Department of Occupational Medicine, Chung Shan Medical University Hospital, Taichung, Taiwan; 3 School of Public Health, National Defense Medical Center, Taipei, Taiwan; 4 School of Respiratory Therapy, College of Medicine, Taipei Medical University, Taipei, Taiwan; 5 Division of Pulmonary Medicine, Department of Internal Medicine, Shuang Ho Hospital, Taipei Medical University, New Taipei City, Taiwan; 6 Department of Internal Medicine, School of Medicine, College of Medicine, Taipei Medical University, Taipei, Taiwan; 7 Institute of Labor, Occupational Safety and Health, Ministry of Labor, New Taipei City, Taiwan; 8 Center of Environmental and Occupational Medicine, Kaohsiung Municipal Hsiaokang Hospital, Kaohsiung, Taiwan; 9 Institute of Biomedical Engineering & Nanomedicine, National Health Research Institutes, Miaoli, Taiwan; 10 Department of Internal Medicine, Division of Cardiology, National Taiwan University Hospital, Taipei, Taiwan; 11 School of Public Health, College of Public Health and Nutrition, Taipei Medical University, Taipei, Taiwan; 12 Department of Public Health, School of Medicine, College of Medicine, Taipei Medical University, Taipei, Taiwan

## Abstract

Occupational exposure to welding fumes causes a higher incidence of cardiovascular disease; however, the association remains unclear. To clarify the possible association, exposure assessment of metal fumes with an aerodynamic diameter of <2.5 μm (PM_2.5_) in welding and office areas was characterized in a shipyard in Taiwan. Cardiovascular toxicity caused by PM_2.5_ was determined in workers (in both the welding and office areas). Significant amounts of bimodal metal fume particles with count median diameters (CMDs) of 14.1~15.1 and 126.3~135.8 nm were produced in the shipyard. Metal fume PM_2.5_ resulted in decreased cell viability and increased levels of 8-hydroxy-2’-deoxyguanosine (8-OHdG), interleukin (IL)-6, and nitric oxide (NO) in human coronary artery epithelial cells (HCAECs). We recruited 118 welding workers and 45 office workers for a personal PM_2.5_ exposure assessment and determination of urinary levels of 8-OHdG, 8-iso-prostaglandin F2α (8-iso-PGF2α), and various metals. We observed that a 10-μg/m^3^ increase in the mean PM_2.5_ concentration was associated with a 2.15% increase in 8-OHdG and an 8.43% increase in 8-iso-PGF2α in welding workers. Both 8-OHdG and 8-iso-PGF2α were associated with Fe and Zn in the urine. In conclusion, metal fume PM_2.5_ could increase the risk of cardiovascular toxicity after inhalation.

The *Occupational Outlook Handbook* published by the US Bureau of Labor Statistics reports that there were about 53,500 Americans employed as welding, soldering, and brazing machine setters, operators, and tenders in 2012^1^. The report shows that a large number of workers are potentially threatened by exposure to metal fumes. Metal fume fever is a flu-like occupational disease caused by the inhalation of metal fumes, which contain such metals as Zn, Mn, Cu, Cd, Ni, and Al, and which leads to respiratory and systemic syndromes that often occur in workers exposed to metal fumes when welding galvanized metal and melting metal^2^,^3^,^4^. Metal fume fever is considered to be a reversible symptom after exposure; however, increasing clinical evidence has found that exposure to metal fumes results in adverse health effects^5^,^6^. For example, workers using an acetylene torch to dismantle galvanized steel in a poorly ventilated area were diagnosed with diffuse alveolar damage to the lungs^5^. The irreversible pulmonary damage may result from repeated exposure to metal fumes (i.e. particulate and gaseous pollutants), which should be further investigated.

Evidence accumulating from epidemiological studies indicates an association between the inhalation of welding fumes and increased incidences of cardiovascular events such as cardiac arrhythmias, myocardial ischemia, and atherosclerosis^7^,^8^. Cavallari and colleagues showed that exposure of boilermaker construction workers to particulate matter with an aerodynamic diameter of <2.5 μm (PM_2.5_) of metal fumes caused alterations in the heart rate variability^9^. Umukoro and colleagues observed that long-term metal particulate exposure is able to decrease cardiac accelerations and decelerations in welding workers^10^. Our previous study showed that the inhalation of occupationally relevant zinc oxide metal fume particles with an aerodynamic diameter of <0.1 μm (PM_0.1_) caused cardiac inflammation and injury to Sprague-Dawley rats^11^. Together, exposure to metal fumes may increase the risk of developing cardiovascular diseases and/or injury; however, these associations remain unclear.

The deposition of welding particles in the airway after inhalation depends on the particle size and morphology as well as the welding methods^12^. Metal fume PM_2.5_ generated by welding processes exists primarily in an oxidized form as aerosolized PM_0.1_ during welding or cutting galvanized sheet metal. PM_0.1_ (so-called nanoparticles) was shown to be able to cross the pulmonary epithelial barrier into the circulation^13^, thereby directly exposing the vascular endothelium to metal fume particles. Cytotoxicity, oxidative stress, and inflammatory responses occur due to metal oxides in human cardiac microvascular endothelial cells and human aortic endothelial cells^14^,^15^,^16^. Inflammation of the endothelium is recognized as playing a central role in the development of atherosclerosis^17^. Shipyards were reported to be important areas of particle exposure in workers^18^. In the present study, we hypothesized that exposure to metal fume PM_2.5_ is associated with cardiovascular toxicity, and that the nature of the response depends on the physicochemistry of the PM_2.5_. First, environmental monitoring was conducted in a shipyard in Taiwan. Metal fume PM_2.5_ was collected from a welding area (which served as a high-exposure group) and an office area (which served as a low-exposure group) in the shipyard for a toxicological evaluation of human coronary artery endothelial cells (HCAECs). Second, a personal PM_2.5_ exposure assessment in welding workers and office workers was conducted. Biomarkers for oxidative stress and cardiovascular diseases, and metals in the urine were determined. Finally, associations of personal PM_2.5_ exposure and urinary metals with the biomarkers were examined.

## Results

### Environmental monitoring

**Figure 1:**
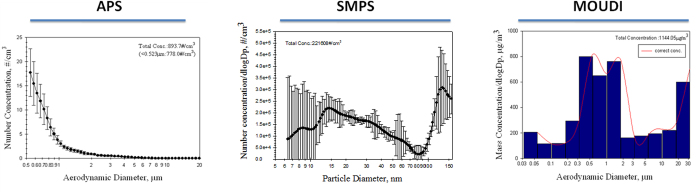
Characterization of profiles of particulate matter with an aerodynamic diameter of <2.5 μm (PM_2.5_) in the welding area between 08:00 and 20:00 on 12~23 August 2013. APS, aerodynamic particle sizer spectrometer; SMPS, scanning mobility particle sizer; MOUDI, micro-orifice uniform deposit impactors.

The profiling of metal fume PM_2.5_ was characterized using the APS and SMPS for the number distribution, and the MOUDI was used for the mass distribution (Fig. 1). APS results showed that 894 particles/cm^3^, ranging 542~19,810 nm, was yielded from welding processes, and the majority of the PM_2.5_ numbers were <1 μm. SMPS results further showed that 221,608 particles/cm^3^ was measured in the range of 5~160 nm with a bimodal distribution, and PM_0.1_ was coagulated when emitted into the atmosphere with a count median diameter (CMD) of 14.1~15.1 nm. Mass concentrations for metal fume PM_10_ (<10 μm), PM_2.5_, and PM_0.1_ were 899, 755, and 81 μg/m^3^, respectively. Ratios of PM_2.5_ to PM_10_ (PM_2.5_/PM_10_) and PM_0.1_ to PM_2.5_ (PM_0.1_/PM_2.5_) were 84% and 11%, respectively. Mass concentrations for office PM_10_, PM_2.5_, and PM_0.1_ were 51, 32, and 5 μg/m^3^, respectively.

### Physicochemical characterization of metal fume PM_2.5_

**Figure 2:**
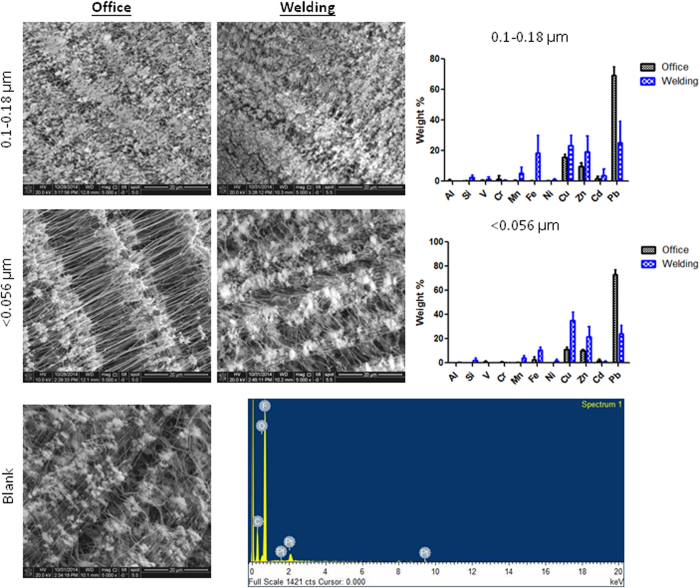
SEM and EDX analyses of metal fume particles that ranged 0.18~0.1 and <0.056 μm collected in the welding and office areas. A blank filter served as the background control. Mn, Fe, Cu, and Zn were higher in the metal fume particles than in office particles.

The physicochemistry of the 0.18~0.1-μm substrate for PM_0.18–1.8_ and the <0.056-μm substrate for PM_0.1_ collected in the welding and office areas during the entire study period was characterized using FE-SEM and EDX (Fig. 2). Generally, the metal fume and office PM_2.5_ were regular in shape and had aggregated. There was a significantly higher amount of metal fume PM_2.5_ collected in the size range of <0.056 μm than the office PM_2.5_. In the size range of 0.18~0.1 μm, Mn, Fe, Cu, and Zn were higher in the metal fume PM_0.18~1.8_ than the office PM_0.18~1.8_. The office PM_0.18~1.8_ was dominated by Pb. Consistently, EDX results showed that the metal fume PM_0.1_ in size was mainly Mn, Fe, Cu, and Zn, whereas the office PM_0.1_ was mainly Pb.

### Cell viability

**Figure 3:**
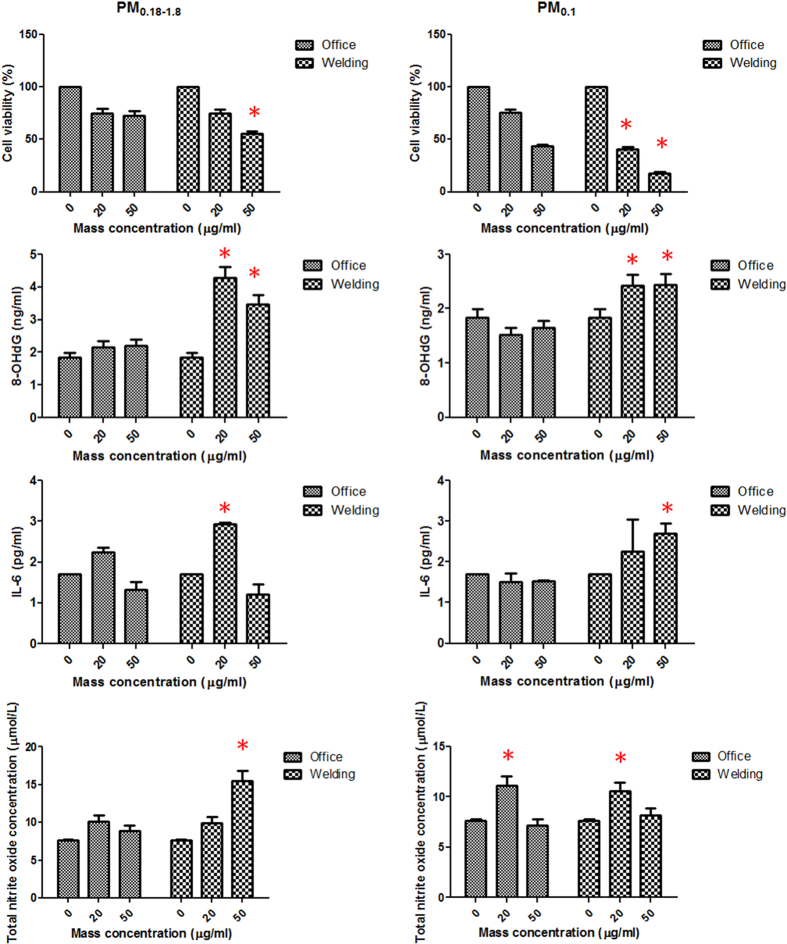
Dose-response relation of cell viability, 8-hydroxy-2′-deoxyguanosine (8-OHdG), interleukin (IL)-6, and nitric oxide (NO) to metal fume particulate matter with an aerodynamic diameter of 0.1~1.8 μm (PM_0.1~__1.8_) and PM_0.1_ in human coronary artery endothelial cells (HCAECs). *p < 0.05.

Figure 3 shows the dose-dependent response for changes in cell viability with PM_0.18~1.8_ and PM_0.1_ exposure. There were significant reductions in cell viability in groups exposed to 20 and 50 μg/ml PM_0.18~1.8_ and PM_0.1_ (*p* < 0.05). Metal fume PM_0.18~1.8_ and PM_0.1_ significantly reduced the viability of HCAECs at 20 and 50 μg/ml compared to the office PM_0.18~1.8_ and PM_0.1_ (*p* < 0.05), except for 20 μg/ml PM_0.18~1.8_.

### 8-OHdG, IL-6, and NO production by HCAECs

Figure 3 shows dose-response relations for 8-OHdG, IL-6, and NO production by HCAECs in response to PM_0.18~1.8_ and PM_0.1_. All of the metal fume PM_0.18~1.8_ and PM_0.1_ at 20 and 50 μg/ml significantly increased the production of 8-OHdG, IL-6, and NO levels compared to the controls (*p* < 0.05), except for 50 μg/ml PM_0.18~1.8_ and 20 μg/ml PM_0.1_ for IL-6 production and 20 μg/ml PM_0.18~1.8_ and 50 μg/ml PM_0.1_ for NO production. When comparing PM_0.18~1.8_ and PM_0.1_ between the welding and office areas, both the 20 and 50 μg/ml metal fume PM_0.18~1.8_ and PM_0.1_ produced higher 8-OHdG levels than did the office PM_0.18~1.8_ and PM_0.1_ (*p* < 0.05). The metal fume PM_0.18~1.8_ and PM_0.1_ produced higher IL-6 and NO levels at 20 or 50 μg/ml exposure than did the office PM_0.18~1.8_ and PM_0.1_ (*p* < 0.05), except for NO production after exposure to welding PM_0.1_.

### Study subjects and exposure assessment

In total, 118 welding workers and 45 office workers were enrolled in this study. Detailed baseline characteristics of the 163 subjects in the study population are presented in Table 1. The majority of the study populations were men among both welding and office workers. The ages of welding workers and office workers were 50.8 ± 10.2 and 48.0 ± 12.0 years, respectively. Their BMIs ranged 17.3~33.3 kg/m^2^. Mean PM_2.5_ concentrations were 48.8 ± 32.3 μg/m^3^ for welding workers and 28.7 ± 15.2 μg/m^3^ for office workers. Welding workers had significantly higher levels of PM_2.5_ exposure than did office workers (*p* < 0.05). The mean temperature and humidity were 22.7~31.1 °C and 54.2~82.8%, respectively, during the study period.

### Urinary 8-OHdG and 8-iso-PGF2α

**Figure 4:**
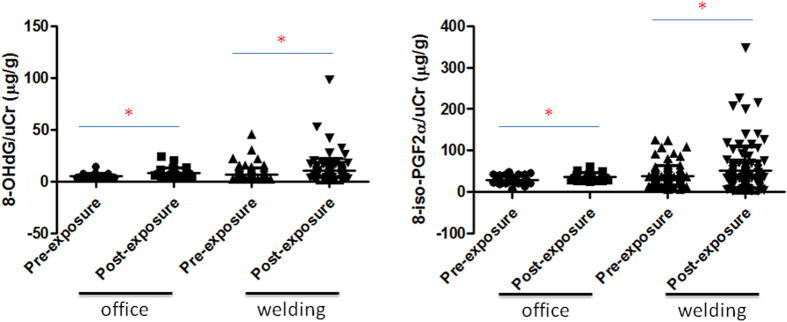
Urinary 8-hydroxy-2′-deoxyguanosine (8-OHdG) and 8-iso-prostaglandin F2α (8-iso-PGF2α) levels after adjusting for urinary creatinine (uCr) in pre- and post-exposure office and welding workers. 8-OHdG and 8-iso-PGF2α levels (adjusted for uCr) in post-exposure office workers and post-exposure welding workers were significantly higher than those in the respective pre-exposure groups. *p < 0.05.

Two biomarkers, 8-OHdG and 8-iso-PGF2α, were used in this study. Levels of 8-OHdG/uCr and 8-iso-PGF2α/uCr were significantly higher in the post-exposure welding and office workers compared to the pre-exposure controls (*p* < 0.05) (Fig. 4). Notably, welding workers had higher levels of 8-OHdG and 8-iso-PGF2α (adjusted with uCr) post-exposure than did office workers.

To determine the associations between the mean PM_2.5_ concentration and urinary markers (8-OHdG and 8-iso-PGF2α), a generalized linear model was used (Table 2). An increase in 10 μg/m^3^ PM_2.5_ was associated with a 2.15% increase in 8-OHdG/uCr (95% confidence interval (CI) = 1.56~2.74, *p* < 0.05) and an 8.43% increase in 8-iso-PGF2α/uCr (95% CI = 2.14~14.72, *p* < 0.05) in welding workers after adjusting for sex, age, the BMI, and smoothing functions of the mean temperature and humidity. There was no significant association of 8-OHdG or 8-iso-PGF2α with PM_2.5_ observed in any workers (welding or office workers).

### Urinary metals

**Figure 5:**
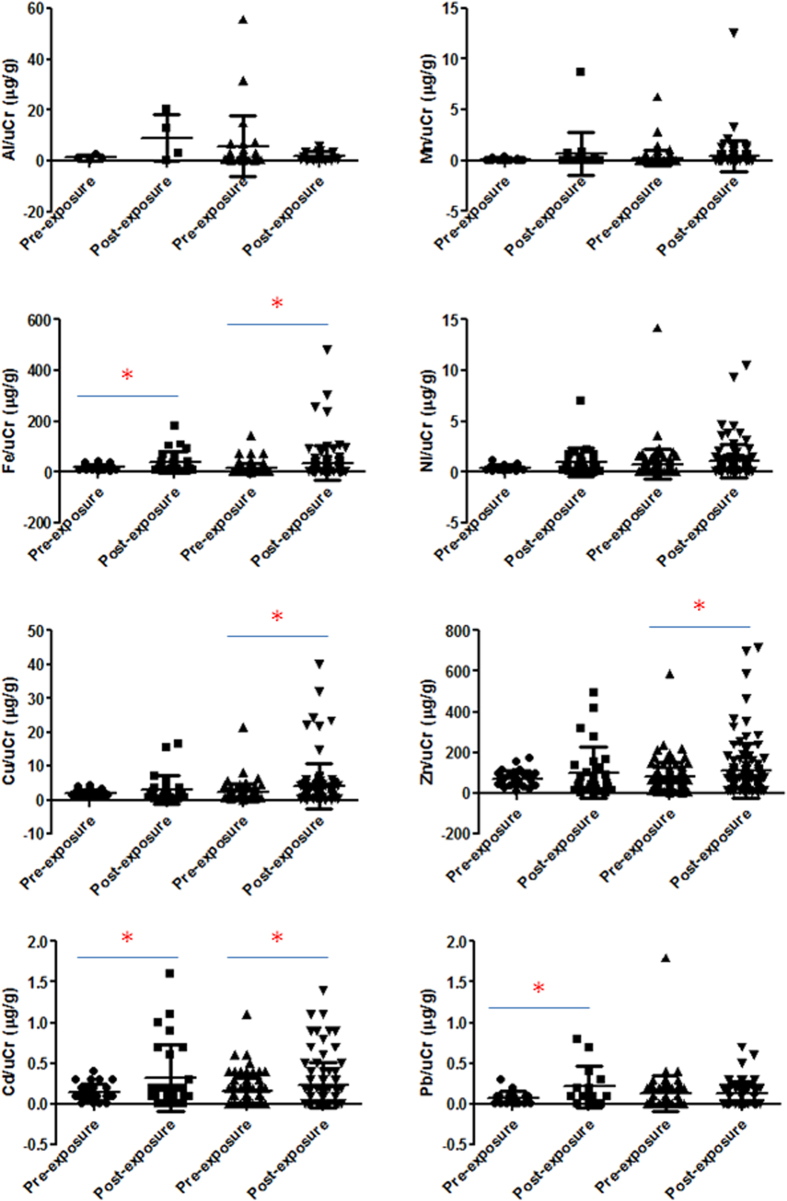
Urinary Al, Mn, Fe, Ni, Cu, Zn, Cd, and Pb levels after adjusting for urinary creatinine (uCr) in pre-exposure office workers, post-exposure office workers, pre-exposure welding workers, and post-exposure welding workers. Fe, Cu, Zn, and Cd were significantly higher in welding workers after exposure compared to their pre-exposure levels (*p < 0.05). Urinary Fe, Cd, and Pb were significant higher in office workers after exposure (*p < 0.05).

After adjusting for uCr, urinary Al, Mn, Fe, Ni, Cu, Zn, Cd, and Pb levels were determined in welding and office workers pre- and post-exposure (Fig. 5). We observed that Fe, Cu, Zn, and Cd were significantly higher in welding workers after exposure compared to pre-exposure levels (*p* < 0.05). Urinary Fe, Cd, and Pb were significantly higher in office workers after exposure (*p* < 0.05).

### Associations of urinary 8-OHdG and 8-iso-PGF2α with metals

Correlations of 8-OHdG and 8-iso-PGF2α with Al, Mn, Fe, Ni, Cu, Zn, Cd, and Pb for welding workers and office workers were determined (Table 3). 8-OHdG was associated with Fe (*r* = 0.167, *p* < 0.05) and Zn (*r* = 0.650, *p* < 0.05). 8-iso-PGF2α was associated with Mn (*r* = 0.280, *p* < 0.05), Fe (*r* = 0.340, *p* < 0.05), Ni (*r* = 0.533, *p* < 0.05), Cu (*r* = 0.513, *p* < 0.05), Zn (*r* = 0.580, *p* < 0.05), Cd (*r* = 0.381, *p* < 0.05), and Pb (*r* = 0.386, *p* < 0.05). Urinary 8-iso-PGF2α had higher associations with urinary Mn, Ni, Cu, Cd, and Pb than did 8-OHdG.

## Discussion

In the present study, the effects of metal fume PM_2.5_ on HCAECs and welding workers were investigated. Four major findings are reported in the present study: (1) significant numbers of PM_0.1_ dominated by Mn, Fe, Cu, and Zn were produced during welding processes; (2) alterations in cell viability, and 8-OHdG, IL-6, and NO levels by the metal fume PM_0.1_ in HCAECs occurred; (3) urinary 8-OHdG and 8-iso-PGF2α levels were significantly higher post-exposure to the metal fume PM_2.5_; and (4) 8-iso-PGF2α was significantly associated with urinary Mn, Ni, Cu, Cd, and Pb levels.

To investigate the potential health impacts caused by exposure to metal fume PM_2.5_, a shipyard was selected in the present study. Our previous study showed that metal fume PM_10_ generated from welding processes in open and semi-open areas in a shipyard were 4~36 and 98~800 μg/m^3^, respectively^19^. Consistently, we observed that the PM_10_ level of metal fumes was 899 μg/m^3^, which suggests that the shipyard is an important site for pulmonary exposure to high levels of metal fume PM_10_. We further observed that the majority of metal fume particles generated from welding processes were predominated by PM_2.5_ for mass concentrations and by PM_0.1_ for number concentrations. The bimodal distribution for the number concentration of the metal fume PM_2.5_ demonstrated that great amounts of PM_0.1_ were generated, emitted into the atmosphere, and rapidly coalesced into larger accumulation-mode particles within nano-sized fractions; however, PM_0.1_ only accounted for 11% of the mass concentration of PM_2.5_. When metal is heated to its melting point, metal oxide fumes are generated. Particle sizes of the generated metal fumes were reported to range 0.1~1.0 μm, and aggregation readily occurs with the formation of larger particles. Previous studies showed that PM_0.1_ is easily transported into the alveolar space through inhalation and may lead to severe health effects due to their physicochemical characteristics^20^. Therefore, we collected metal fume and office PM_2.5_ for physicochemical characterization. In the present study, two filter substrates were used: 0.18~0.1 μm for PM_0.1~__2.5_ and <0.056 μm for PM_0.1_. We observed that aggregation was commonly present in the metal fume and office PM_2.5_. Among these particles, Mn, Fe, Cu, and Zn dominated in the metal fume PM_2.5_ (0.18~0.1 and <0.056 μm). Notably, the office PM_2.5_ (0.18~0.1 and <0.056 μm) contained higher percentages of Pb, which could have resulted from cigarette smoking in the office area.

To investigate the toxicity of metal fume PM_2.5_ at the cellular level, HCAECs were exposed to two different size fractions (0.18~0.1 and <0.1 μm) collected from the welding and office areas. The endothelium is a monolayer of cells constituting an interface between the blood and vascular walls, which plays an important role in physical and biological protection of vasoactive function and homeostasis. Also, cells that we used in this study are crucially involved in regulating coronary blood flow and cardiac functions and are consequently useful for *in vitro* studies of cardiovascular diseases. Previous studies showed that oxidative-inflammatory reactions of the endothelium are recognized as playing central roles in the development of cardiovascular disease^17^. We observed that oxidative stress, inflammation, and NO were significantly increased in HCAECs by welding PM_2.5_ compared to office PM_2.5_, particularly the smaller size fraction of PM_0.1_. We observed that welding PM_0.1_ had higher bioreactivity than welding PM_0.18~1.8_ in HCAECs based on mass metrics, which may be attributed to the particle numbers, surface areas, and chemical compounds in the particles. Endothelium-derived NO is an essential regulator of cardiovascular homeostasis and immune responses^21^. Consistent with our findings, previous studies showed that metal oxide nanoparticles caused significant cell death and elevated inflammatory responses in human aortic endothelial cells and NO production in rats^16^,^22^. Because of the importance of endothelial inflammation in the development of cardiovascular pathology, based on our findings, we suspect that occupational exposure to welding fume PM_2.5_ induces an oxidative-inflammatory response. Also, the different oxidative-inflammatory responses between PM_0.18~1.8_ and PM_0.1_ may be associated with their unique physicochemical characteristics.

Next, we recruited 163 subjects from the office and welding areas in the shipyard to investigate adverse health effects caused by metal fume PM_2.5_ exposure. The mass and number particle distributions and chemical profiles in welding and office workplaces were characterized in the present study. We then conducted personal PM_2.5_ exposure assessments for the 163 subjects, which showed that welding workers were exposed to significantly higher levels of PM_2.5_ than were office workers during work time. Generally, the personal exposure to PM_2.5_ in welding workers was significantly lower (48.8 μg/m^3^) than the U.S. Occupational Safety and Health Administration (OSHA) permissible exposure limits (PELs) for respirable fraction particles (5 mg/m^3^) in the present study^2^. However, we still observed significant increases in levels of urinary 8-OHdG and 8-iso-PGF2α in welding and office workers post-exposure. Our observations are consistent with previous findings in a control human exposure study^23^. Furthermore, we found that a 10-μg/m^3^ increase in the mean PM_2.5_ resulted in a 2.15% increase in 8-OHdG/uCr and a 8.43% increase in 8-iso-PGF2α/uCr in welding workers. The correlation suggests that occupational exposure to PM_2.5_ could be an important health concern in welding workers. 8-OHdG is produced due to a hydroxyl radical attack at the C-8 position of deoxyguanosine in DNA, leading to oxidative DNA damage. Previous studies showed that urinary 8-OHdG is a biomarker for evaluating the extent of repair of oxidative stress-induced DNA damage in clinical and occupational settings^24^,^25^. For example, an increase in 8-OHdG in boilermakers was observed after exposure to high levels of metal-containing particles^26^. Importantly, we observed that urinary 8-iso-PGF2α levels were significantly related to PM_2.5_ exposure in welding workers. Urinary 8-iso-PGF2α is considered a biomarker for assessing cardiovascular diseases, such as coronary heart disease^27^. These welding workers who were exposed to metal fume PM_2.5_ not only had increased oxidative stress post-exposure, but also may have had increased cardiovascular toxicity. Notably, increases in urinary 8-OHdG and 8-iso-PGF2α in welding and office workers after exposure could have resulted from exercise^28^, which should be considered in future studies.

Urinary 8-OHdG has been linked to pulmonary exposure to V, Mn, Ni, and Pb in PM_2.5_ in boilermakers^26^, suggesting that PM_2.5_-bound metals may elevate oxidative stress in workers. In the present study, we found that Mn, Fe, Cu, and Zn were dominant in the metal fume PM_2.5_, whereas Pb was dominant in the office PM_2.5_. Furthermore, we observed that Fe, Cr, Zn, and Cd were highly excretable in urine after exposure to metal fume PM_2.5_. To determine associations between these heavy metals and adverse health effects, urinary 8-OHdG and 8-iso-PGF2α levels were correlated with eight urinary metals. We observed that 8-iso-PGF2α was more sensitive to these metals (except for Al) than was 8-OHdG. Both 8-OHdG and 8-iso-PGF2α were associated with Fe and Zn in the urine. Heavy metals are considered causative agents of reactive oxygen species formation^29^. Some metals, such as Fe, are capable of redox cycling and generate superoxides and hydroxyl radicals through the Fenton reaction^30^,^31^. Our findings suggest that heavy metals in metal fume PM_2.5_ play critical roles in regulating oxidative stress and cardiovascular toxicity.

Some limitations of the present study should be considered: (1) parallel environmental monitoring should have been conducted in the office area, which would have clarified possible contamination resulted from the welding area; (2) personal PM_0.1_ assessment was not carried out due to the limitations of the instruments; and (3) the low-exposure group selected may have been exposed to other indoor air pollutants such as cigarette smoke that may have increased urinary metals and biomarkers.

## Conclusions

In accordance with results from HCAECs and welding workers, we demonstrated an association of metal fume PM_2.5_ with alterations in biomarkers. In the present study, personal protective equipment was used during all welding processes. However, increased 8-OHdG and 8-iso-PGF2α levels in the urine were still observed. These observations suggest that increasing ventilation and reducing exposure times may be required for occupational health protection. Investigation of the underlying mechanisms and functional parameters (such as electrocardiography) in metal fume PM_2.5_-induced cardiovascular disease is required in future work.

## Materials and Methods

### Environmental monitoring

To evaluate occupational concentrations of PM_2.5_, PM_2.5_ collection and 12-h continuous measurements were conducted between 08:00 and 20:00 on Monday to Friday during 12~23 August 2013 in a shipyard located in southern Taiwan. A semi-open area where welding of galvanized metal occurred was selected for the exposure assessment. Tungsten inert gas (TIG) welding was the main method used by this company. A TSI aerodynamic particle sizer spectrometer (APS; model 3321, TSI, USA) and a TSI scanning mobility particle sizer with nano-DMA (SMPS; model 3936, TSI) were used in parallel to monitor the size distribution of the metal fume PM_2.5_ in the welding and office areas, with ranges of 542~19,810 and 5~160 nm, respectively. The APS and SMPS were calibrated using 100-nm NIST-traceable PSL standard particles before the experiment. Micro-Orifice Uniform Deposit Impactors (MOUDIs; MSP, USA), which were used for PM_2.5_ collection onto Teflon substrates, were set up along with the APS and SMPS in the same welding and office areas with a constant flow rate of 30 l/min. The MOUDI was used to size the PM, ranging 0.056~18 μm in 50% cut-off diameters (18, 10, 5.6, 2.5, 1.8, 1.0, 0.56, 0.32, 0.18, 0.1, and 0.056 μm), using 11 inertial-based cascade impactors^32^.

### Physicochemical characterization

The physicochemistry of the 0.18~0.1-μm substrate for PM_0.18~1.8_ and the <0.056-μm substrate for PM_0.1_ collected using the MOUDI on Monday to Friday during 12~23 August in the welding and office areas was characterized. The preparation and analytical processes for field emission-scanning electron microscopy (FE-SEM) were previously reported^33^. An FE-SEM (JEOL 2100, Jeol, Japan) and an energy-dispersive x-ray (EDX) microanalysis were used to investigate physicochemical characteristics of the PM_2.5_. The FE-SEM was operated at an accelerating voltage of 15 kV and a 2.5-μm spot size. Elemental analysis was performed using the EDX Genesis Microanalysis System.

### Culture of human coronary artery endothelial cells (HCAECs) and treatment

HCAECs obtained from Lonza (Basel, Switzerland) were cultured in HCAEC growth medium (Lonza) in an incubator with 95% humidified air and 5% CO_2_ at 37 °C; only cells in passage 5 were used for exposure^32^. HCAECs were seeded onto surface-coated transwells at a density of 10^5^ cells/ml for 24 h. PM_2.5_ samples collected from the welding and office areas were removed from the Teflon substrates according a previous report^34^, and the substrates were pooled together into two size fractions: PM_0.18~1.8_ (0.18~1.8 μm) and PM_0.1_. The metal fume PM_0.18~1.8_ and PM_0.1_ samples were prepared at 0, 20, and 50 μg/ml with cell media for a 4-h exposure in cells at 37 °C in a 5% CO_2_ humidified atmosphere. Each experiment was run in quadruplicate. Concentrations of particles were chosen to produce a 50% reduction in cell viability according to previously described criteria^35^.

### Cell viability

Cell viability was examined by the trypan blue dye exclusion assay. Dead and viable cells were counted using a hemocytometer with the aid of an inverted light microscope (Nikon eclipse Ti, USA). Cells were counted under a microscope in four 1 × 1-mm squares of one chamber, and the average number of cells per square was determined. Cell counting was done in triplicate. Viability was expressed as a percentage (%) of surviving cells counted.

### 8-Hydroxy-2’-deoxyguanosine (8-OHdG), interleukin (IL)-6, and nitric oxide (NO) *in vitro*

Enzyme-linked immunosorbent assay (ELISA) kits were used to determine concentrations of 8-OHdG (JaICA, Japan), IL-6 (R&D Systems, USA), and NO (determined as nitrite concentration; R&D Systems) in cell supernatants after exposure, following the manufacturer’s instructions.

### Study population and personal PM_2.5_ exposure assessments

The study protocol was approved by the Ethics Committee of the Taipei Medical University-Joint Institutional Review Board (Taipei, Taiwan). Methods were carried out in accordance with approved guidelines. All subjects received written and oral information prior to inclusion and provided informed consent. This human study was designed to investigate associations between personal PM_2.5_ exposure with levels of urinary 8-OHdG, 8-iso-prostaglandin F2α (8-iso-PGF2α), and metals among our study participants from the shipyard. In total, 118 welding workers and 45 office workers were recruited for this study. The exclusion criteria for participants were those who had cardiovascular diseases or a history of cardiovascular diseases, such as coronary artery disease, arrhythmias, hypertension, diabetes mellitus, and dyslipidemia. Urine samples from each worker were collected at two time points: at the beginning (Monday; pre-exposure; baseline for 1-week exposure) and end of the work week (Friday; post-exposure; 1-week exposure). Personal exposure to PM_2.5_ was measured for each worker from 08:00 and 17:00 on 19~23 August 2013 using two real-time dust monitors (DUST-check Portable Dust Monitor model 1.108, Grimm Labortechnik, Ainring, Germany). We assigned two technicians carrying dust monitors to accompany each worker for 10 min per hour to measure personal PM_2.5_ exposure while working. The exposure assessment was conducted on approximately 100 workers per day during the study period (each worker 1 time per day and at least 3 times per week). Average 10-min/h mass concentrations of PM_2.5_, temperature, and relative humidity were monitored by the dust monitor and summarized to the mean PM_2.5_ for each worker for the statistical analysis. Also, the age, sex, body-mass index (BMI), medications, and working characteristics (job title, years of work experience, time of work, use of personal protective equipment, etc.) were obtained from workers by a questionnaire. Study subjects (welding) were provided with masks (non-woven fabric). Higher levels of protective equipment were provided for specific workplaces.

### Urinary 8-OHdG and 8-iso-PGF2α

Two urine samples were collected from each worker on Monday morning (at around 08:00) and Friday afternoon (at around 17:00). An ELISA was used to determine urinary 8-OHdG (JaICA) and 8-iso-PGF2α levels (Abcam, UK), according to the manufacturer’s instructions. Levels of 8-OHdG and 8-iso-PGF2α were adjusted with the urinary creatinine (uCr) level.

### Urinary metal concentrations

Eight metals in the urine were determined as previously described^36^. Briefly, urinary samples were digested using concentrated nitric acid (Fisher Scientific, USA) in a MARS 5 microwave system (CEM, USA) in advanced Teflon-lined composite vessels (CEM), followed by 0.45-μm polyvinylidene difluoride filtration (ChromTech, USA). Nitric acid and deionized water (>18 MΩ) were added to the samples for a final concentration of 5% nitric acid. Inductively coupled plasma-mass spectrometry (ICP-MS; Agilent 7500, USA) was used to determine the following eight metal concentrations in urinary samples: Al, Mn, Fe, Ni, Cu, Zn, Cd, and Pb. Deionized water blanks and a certified rock standard (BCR1) were used to detect contamination and accuracy of the analyses. The relative percentage difference was <10%. Levels of metals were adjusted using the uCr level.

### Statistical analysis

The Shapiro-Wilk test was used to test for normality. For comparisons among multiple values, a one-way analysis of variance (ANOVA) with Tukey’s post-hoc test was used. For comparisons between groups, Student’s *t*-test was used for the significance analysis. A paired *t*-test was used to compare PM_2.5_ concentrations, meteorological conditions, and urinary biomarkers. The outcome variables were 8-OHdG and 8-iso-PGF2α, and the exposure variables were the mean PM_2.5_. Sex, age, BMI, work (welding vs. office), years of work experience, mean temperature, and mean humidity were adjusted for in all models. Pollution effects are expressed as percent changes by 10-μg/m^3^ changes as [β × 10 ÷ M] × 100% for urinary markers, where β and M are the estimated regression coefficient and the mean of each marker, respectively. Pearson’s correlation coefficient was used to evaluate relations among urinary metals, 8-OHdG/uCr, and 8-iso-PGF2α/uCr. The level of significance was set to *p* < 0.05. Values in figures are expressed in the mean ± standard deviation (SD).

## Additional Information

**How to cite this article**: Lai, C.-Y. *et al*. Physicochemistry and cardiovascular toxicity of metal fume PM_2.5_: a study of human coronary artery endothelial cells and welding workers. *Sci. Rep.* **6**, 33515; doi: 10.1038/srep33515 (2016).

## Figures and Tables

**Table 1:** Basic characteristics, personal exposure to particulate matter with an aerodynamic diameter of <2.5 μm (PM_2.5_), and meteorological conditions of the 163 study subjects in the shipyard.

Variable	Welding workers (*N* = 118)	Office workers (*N* = 45)	*p* value
Sex (no.)			
Women	1	1	—
Men	117	44	—
Smoking (no.)			
Current	28	10	—
Never	90	35	—
Age (years)			
Mean	50.8 ± 10.2	48.0 ± 12.0	0.153
Range	22~64	24~64	
Body mass index (kg/m^2^)			
Mean	24.0 ± 2.9	24.1 ± 2.5	0.924
Range	17.3~33.3	18.6~30.8	
PM_2.5_ (μg/m^3^)^1^			
Mean	48.8 ± 32.3	28.7 ± 15.2	0.021*
Range	29.5~78.4	15.4~36.6	
Temperature (°C)^1^			
Mean	28.5 ± 1.6	24.9 ± 1.1	0.114
Range	26.3~31.1	22.7~27.5	
Humidity (%)^1^			
Mean	67.3 ± 7.3	61.8 ± 4.6	0.072
Range	60.3~82.8	54.2~66.2	

^1^Average 10-min/h mass concentrations of PM_2.5_, temperature, and relative humidity (each worker 1 time per day and at least 3 times per week).

^*^p < 0.05.

**Table 2:** Percentage changes (95% confidence interval (CI)) in urinary 8-hydroxy-2′-deoxyguanosine (8-OHdG)/urinary creatine (uCr) and 8-iso-prostaglandin F2α (8-iso-PGF2α)/uCr for 10 μg/m^3^ increase in mean concentration of particulate matter with an aerodynamic diameter of <2.5 μm (PM_2.5_).

	All workers (*N* = 163)	Welding workers (*N* = 118)	Office workers (*N* = 45)
8-OHdG/uCr	1.24	2.15*	1.88
	(0.89, 1.59)	(1.56, 2.74)	(0.99, 2.77)
8-iso-PGF2α/uCr	3.26	8.43*	0.89
	(0.61, 5.91)	(2.14, 14.72)	(−1.27, 3.05)

Coefficients are expressed as percent changes for a 10-μg/m^3^ increase in mean PM_2.5_ in models adjusting for sex, age, body mass index and smoothing functions of mean temperature and humidity.

^*^p < 0.05.

**Table 3:** Correlations of eight urinary metals with 8-hydroxy-2′-deoxyguanosine (8-OHdG) and 8-iso-prostaglandin F2α (8-iso-PGF2α) in the 163 study subjects.

	8-OHdG/uCr	8-iso-PGF2α/uCr
Al	0.184	0.027
Mn	0.053	0.280*
Fe	0.167*	0.340*
Ni	0.083	0.533*
Cu	0.062	0.513*
Zn	0.650*	0.580*
Cd	0.148	0.381*
Pb	0.142	0.386*

^*^p < 0.05.

uCr, urinary creatine.
